# Prime detection of *Dirofilaria immitis*: understanding the influence of blocked antigen on heartworm test performance

**DOI:** 10.1186/s13071-018-2736-5

**Published:** 2018-03-20

**Authors:** Susan Little, Meriam Saleh, Megan Wohltjen, Yoko Nagamori

**Affiliations:** Department of Veterinary Pathobiology, Center for Veterinary Health Sciences, Oklahoma State University, Stillwater, OK USA

**Keywords:** Antigen, Diagnosis, *Dirofilaria immitis*, False negative, False positive, Heartworm, Heat treatment

## Abstract

Detection of circulating antigen of *Dirofilaria immitis* has been a mainstay of identifying heartworm infection in clinical practice for the past three decades. Several validated commercial antigen tests have very good sensitivity, specificity, and positive predictive values, especially when used in patients for which heartworm infection is likely. In some dogs and cats infected with heartworm, antigen may not be available for detection although present in the patient sample; heat pretreatment of these samples reveals the antigen, changing the false negative to positive. This phenomenon was documented in the literature in the 1980s but subsequently overlooked by the heartworm research community for many years. In this review, we provide a summary of the current understanding of the role of heat reversal in diagnosing heartworm infection. This additional diagnostic step is most important for patients in which heartworm infection is likely, such as dogs or cats in an endemic area with an inconsistent history of heartworm preventive use, or dogs with a prior diagnosis of heartworm infection that were recently treated. To illustrate the concept, we share a summary of results from canine samples tested at the state veterinary diagnostic laboratory in Oklahoma, USA in 2017 by modified Knott test and by commercial antigen test before and after heat treatment of samples; in this sample set, heat treatment changed all *D. immitis* microfilaria-positive but antigen-negative samples to antigen-positive. Pet dogs with a history of consistent preventive use are unlikely to become positive with heat pretreatment; for that reason, routine pretreatment of all samples tested in a veterinary practice is not recommended. We also review known causes of false negative and false positive results on heartworm antigen tests that, although uncommon, can complicate accurate diagnosis in individual patients. Together, this review provides a primer to aid understanding of strategies that can enhance accurate diagnosis of heartworm infection in veterinary practice and clinical research.

## Background

Diagnosis of heartworm infection in clinical practice relies upon detecting antigen of *Dirofilaria immitis* in serum, plasma, or whole blood samples from canine and feline patients. Historically, microfilaria tests were also widely used to test dogs; however, in most canine surveys antigen assays detect more infections than tests for microfilariae (Table 1). The greater sensitivity of antigen tests; the presence of naturally occurring, amicrofilaremic, or occult, infections; and the microfilaricidal effects of macrocyclic lactone-based heartworm preventives led to the perception that screening for microfilariae had limited clinical value [1, 2]. From 1992–2012, American Heartworm Society guidelines stated that less than 1% of microfilaremic dogs test antigen-negative [3, 4], a perception apparently based on data from the 1980s when antigen tests were usually performed on pre-treated samples [5–7]. Due in part to recent data on blocked antigen causing false negative antigen test results, both the American Heartworm Society and the Companion Animal Parasite Council currently recommend testing all dogs using both a microfilaria test and an antigen test [8, 9].

**Table 1:** Canine surveys comparing prevalence of *Dirofilaria immitis* (*Di*) infection by detection of antigen (Ag) using commercial assays without pre-treatment of samples and detection of microfilaria (MF) by microscopy or PCR

Country	*Di* Ag + (%)	*Di* MF by microscopy (%)	*Di* MF by PCR (%)	Other MF detected (%)	Reference
Italy	65/630 (10.3)	79/630 (12.5)	NR	12/630 (0.8)^a^; 76/630 (9.2)^b^	[13]
Portugal	65/696 (9.4)	84/696 (12.1)	40/41 (97.6)	3/696 (0.4)^b^	[14]
Portugal	78/304 (25.7)	61/304 (20.1)	NR	NR	[15]
Romania	16/194 (8.2)	11/194 (5.7)	11/24 (45.8)	12/194 (6.2)^c^	[16]
Australia	48/404 (11.9)	23/404 (5.7)	NR	15/404 (3.7)^a^	[24]
Brazil	8/611 (1.3)	6/611 (1.0)	NR	42/611 (6.9)^a^	[25]
USA	45/616 (7.3)	26/616 (4.2)	NR	6/616 (1.0)^a^	[31]
Costa Rica	16/146 (11.0)	17/146 (11.6)	17/33 (51.5)	16/146 (11.0)^a^	[48]
Greece	28/750 (3.7)	19/750 (2.5)	NR	10/750 (1.3)^a^; 17/750 (2.3)^c^	[54]
USA	31–34/110 (28.2–30.9)	18/110 (16.4)	NR	1/110 (0.9)^a^	[55]
USA (Tennessee)	93/673 (13.8)	213/3608 (5.9)	NR	NR	[56]
South Korea	36/127 (28.3)	13/127 (10.2)	NR	NR	[57]
Dominican Republic	18/104 (17.3)	14/104 (13.5)	NR	NR	[58]
Haiti	55/210 (26.2)	NR	46/207 (22.2)	3/207 (1.4)^a^	[59]

^a^Microfilaria of *Acanthocheilonema reconditum* detected

^b^Microfilaria of *Acanthocheilonema dracunculoides* detected

^c^Microfilaria of *Dirofilaria repens* detected

*Abbreviation*: NR, not reported.

The antigen targeted by the various commercial assays is primarily secreted by adult female heartworms; although all stages of *D. immitis* produce some antigen, the amount of circulating antigen present is considered largely related to the number and age of the female heartworms present in the animal [1, 5]. Although false positive results have been reported (see discussion below), commercial antigen tests are considered to be very specific, with most assays described with a specificity at or approaching 100%. In contrast, the sensitivity of the different tests can vary widely in dogs and cats (Table 2, 3). This variance is usually attributed to the platform for a given antigen test, test performance characteristics, and the age and number of female heartworms present in the patients from which samples were collected [2, 10, 11]. The microtiter plate enzyme-linked immunosorbent assay (ELISA) is considered the most sensitive platform and detects antigen in as many as 85.7% of dogs infected with a single adult female worm; reported sensitivity for microtiter plate ELISA when at least 3 adult female worms are present is 100% [1]. Many of the lateral flow immunochromatographic assays and the membrane-bound ELISAs, which are designed to provide rapid in-clinic results, also demonstrate good sensitivity and can detect antigen in 46–76.2% of patients infected with a single female worm and 84–100% of patients with 3 or more female worms [1, 2, 10]. Repeated evaluation of the same test(s) can yield different performance characteristics due to differences in specimens included in the analysis, test kit version, procedures used, and method of heartworm infection verification [12].

**Table 2:** Reported performance characteristics of selected commercial heartworm antigen tests used in dogs

Test	% Sensitivity (number of live adult female heartworms)	% Specificity	Heartworm positive/total tested	Reference
DiroCHEK®	85.7 (1), 95 (2), 100 (≥ 3)	100	108/208^b, c, d, e^	[1]
PetChek® HTWM PF	76.2 (1), 85 (2), 100 (≥ 3)	100		
Solo Step® CHBatch	71.4 (1), 95 (2), 96.5 (≥ 3)	100		
Solo Step® CH	76.2 (1), 95 (2), 96.5 (≥ 3)	100		
ICT Gold® HW	61.9 (1), 85 (2), 93 (≥ 3)	100		
SNAP® Heartworm PF	76.2 (1), 85 (2), 100 (≥ 3)	100		
Witness® HW	71.4 (1), 90 (2), 94.7 (≥ 3)	100		
AbboScreen™	71.4 (1), 100 (2), 93 (≥ 3)	96		
FILARIA IC (Italian)	76.2 (1), 85 (2), 100 (≥ 3)	100		
Witness™ HW (Australian)	66.7 (1), 90 (2), 94.7 (≥ 3)	100		
VetScan CHAT	78	97	208/240^b, e^	[2]
SNAP® Heartworm RT	84	97		
Solo Step® CH	79	97		
PetChek® HTWM PF	45 (0^a^), 77 (1–2), 94 (≥ 3)	97	140/237^e^	[10]
DiroCHEK®	40 (0^a^), 71 (1–2), 94 (≥ 3)	94		
SNAP® Heartworm PF	35 (0^a^), 65 (1–2), 94 (≥ 3)	98		
Solo Step® CH	35 (0^a^), 56 (1–2), 90 (≥ 3)	98		
AbboScreen	30 (0^a^), 46 (1–2), 84 (≥ 3)	96		
VetScan VS2	33	100	40/90^e^	[11]
SNAP® Heartworm RT	90	100		
SNAP® Heartworm RT	90.9	98.8	84/150^b, f^	[37]
Witness® Heartworm	97.0	96.4		
Filarchek	97.6	100	41/107	[60]
VetScan	92	100	25/49^e^	[61]
DiroCHEK®	100 (≥3)	100	NR	[62]
Witness® Heartworm	97.7	99.3	134/285^e, f^	[63]

^a^Not infected or infected only with male, immature, or dead worms

^b^Heartworm infection established naturally

^c^Heartworm infection established by subcutaneous injection of third-stage larvae

^d^Heartworm infection established by surgical transplantation

^e^Infection verified by necropsy

^f^Infection verified by comparison to DiroCHEK®

**Table 3:** Reported performance characteristics of selected commercial heartworm antigen tests used in cats

Test	% Sensitivity	% Specificity	Heartworm positive/total tested	Reference
VetScan	79.9	99.7	29/380^a^	[61]
DiroCHEK®	89.7	100	39/81^a, b, c, d^	[64]
SNAP® Feline Triple®	89.3	99.5	26/238^a, e^	[65]
DiroCHEK®	78.9	98.1	19/330^a, b^	[66]
SNAP® Feline HTWM	73.7	99.4		
SNAP® Feline HTWM	79.3	98.0	29/380^a, b^	[67]
CHAT Canine HTWM	79.3	99.7		
DiroCHEK®	86.2	99.1		

^a^Infection verified by necropsy

^b^Heartworm infection established naturally

^c^Heartworm infection established by subcutaneous injection of third-stage larvae

^d^Heartworm infection established by surgical transplantation

^e^Infection verified by comparison to PetChek® HTWM PF

## Discordant results

Because of the increased sensitivity of antigen tests over microscopic detection of microfilariae alone, most surveys of naturally infected dogs that include both approaches document that antigen detection identifies more heartworm infections (Table 1). However, in some populations, a surprising number of samples are antigen-negative but microfilaria-positive. In comparing reported results from dogs with evidence of heartworm infection, 6.0–38.7% of dogs with *D. immitis* microfilariae were antigen-negative when tested [13–16]. This particular discordant result can be difficult to understand intuitively. If adult heartworms are present in a dog, mating, and producing a high enough level of microfilaremia to be recognized by microscopy, then adequate antigen should be available for detection. However, in most surveys, some dogs have microfilariae of *D. immitis* but remain antigen-negative even when the most sensitive antigen detection assays are used (Table 4).

**Table 4:** Results from *Dirofilaria immitis* (*Di*) antigen (Ag) and microfilariae (MF) tests on dogs for naturally occurring heartworm infection. In each study listed, microfilariae were confirmed as *Dirofilaria immitis* by acid phosphatase stain or by PCR unless otherwise noted

No. of dogs (% positive)^a^	*Di* Ag + *Di* MF + (%)	*Di* Ag + *Di* MF - (%)	*Di* Ag - *Di* MF + (%)	*Di* Ag - *Di* MF - (%)	Reference
630 (16.2)^b^	42 (6.7)	23 (3.7)	37 (5.9)^b^	555 (88.1)	[13]
696 (15.1)	49 (7.0)	16 (2.3)	41 (5.9)	591 (84.9)	[14]
304 (27.3)	56 (18.4)	22 (7.2)	5 (1.6)	221 (72.7)	[15]
24 (62.5)	6 (25.0)	4 (16.7)	5 (20.8)	9 (37.5)	[16]
404 (12.1)	21 (5.2)	25 (6.2)	3 (0.7)	355 (87.9)	[24]
616 (7.6)	24 (3.9)^c^	21 (3.4)	2 (0.3)^b, c^	569 (92.4)	[31]
104 (18.3)	13 (12.5)	5 (4.8)	1 (1.0)	85 (81.7)	[58]

^a^Positive for *Dirofilaria immitis* by at least one laboratory method

^b^Inferred from text description of reported results and prior to heat treatment of samples

^c^Microfilariae identified by morphology alone

Potential explanations for failing to detect antigen in dogs with circulating microfilariae include misidentification of microfilariae, death of adult worms with persistence of microfilariae, and transfusion of microfilaremic blood or transplacental transmission from a microfilaremic dam to her pups [17, 18]. Microfilariae other than *D. immitis* commonly found in canine blood include *Acanthocheilonema reconditum*, *A. dracunculoides* and *D. repens* [19]. Laboratory confirmation of microfilaria as *D. immitis* and careful review of the history can explain some of the discordant results between microfilaria testing and antigen testing, and inherent limitations of test sensitivity may explain the remaining discrepancies. An antigen test which is 85–90% sensitive would be expected to miss approximately 10–15% of infections in practice; when confidence intervals are included, the range of potential missed infections is wider. Blocked antigen is a more recently re-identified potential cause of false negative antigen test results and one which can be addressed by pre-treating the sample prior to testing. By freeing antigen trapped in immune complexes, detection in some patients is improved. Heat pretreatment, with or without addition of EDTA, is a common method for disrupting immune complexes in many systems; dissociation of complexes may also be achieved using pepsin or an acidic pH [20–22].

### Unexpectedly negative antigen tests

Although commercial antigen tests play a critical role in detecting *D. immitis* infections, some data suggest that relying on antigen tests alone and forgoing efforts at microfilaria detection can result in failing to identify heartworm infection in some patients. For example, using two different commercial assays, antigen was not detected in 21.6% or 24% of serum samples from dogs from Argentina with *D. immitis*-confirmed microfilaremia [23], and 38.7% of microfilaremic dogs in Portugal with PCR confirmed *D. immitis* microfilaremia did not have detectable antigen present [14]. Antigen tests also sometimes fail to detect infection in dogs with adult *D. immitis* confirmed at necropsy. In Australia, 8/15 (53.3%) dogs with adult *D. immitis* recovered were antigen-negative, and 6/14 (42.9%) confirmed heartworm infected dogs in Brazil were antigen-negative [24, 25].

A possible explanation for many of these antigen-negative discordant results in dogs confirmed infected by either detection of microfilariae or recovery of adult heartworms at necropsy is the presence of blocked antigen [5, 26]. In some patients infected with heartworm, antigen is present in circulation but apparently trapped in immune complexes, preventing detection on commercial assays; pre-treating serum or plasma samples to disrupt the immune complexes and then repeating the test changes these false negative antigen tests to true positive [26, 27]. Pretreatment of samples to disrupt immune complexes was routinely practiced when antigen tests for *D. immitis* first became available [5–7]; diagnostic labs often pre-treat samples prior to running antigen assays for other pathogens including fungi (e.g. *Histoplasma* sp., *Aspergillus* sp.), viruses (HIV, dengue), protozoal agents (e.g. *Leishmania* sp.), and others [20–22].

### Heat reversal to support heartworm diagnosis

Heat treatment of samples prior to running heartworm antigen tests has been demonstrated to result in increased detection of antigen in samples from both dogs and cats in a number of different studies in recent years. These reports include samples from both experimentally infected, necropsy-confirmed *D. immitis* infections and natural infections. Evaluation of samples collected from cats 196 days (6.5 months) and 224 days (7.5 months) after experimental infection with third-stage larvae and confirmed to harbor adult heartworms at necropsy demonstrated that 5/6 (83.3%) changed from false negative to true positive after heat pretreatment; one cat was antigen-positive with heat pretreatment as early as day 168 (5.6 months) [28]. These data suggest cats may be particularly likely to have blocked antigen, especially early in infection when antibodies are at peak levels. Similarly, heat pretreatment of samples from dogs experimentally infected with third-stage larvae and confirmed to have adult heartworms at necropsy has been shown to improve and allow earlier detection of antigen. In one study, antigen was not detected in canine samples collected 128 days (4.3 months) after infection, but after heat pretreatment, all (8/8) were positive. Evaluation of samples collected 150–152 days (5 months) after infection showed 6/14 (42.9%) were positive before heat pretreatment but all (14/14) were positive after heat pretreatment [29]. Another study showed that heat pretreatment of samples allowed detection of antigen in experimentally infected dogs an average of one month earlier than when using unheated samples [30].

In surveys of dogs from animal shelters in the United States for naturally occurring heartworm infection, 11/154 (7.1%) and 29/558 (5.2%) canine samples changed from negative to positive on microtiter well based ELISA assays after pretreatment of samples by heating [27, 31]. Furthermore, when antigen-positive samples were subjected to pretreatment by heating, the optical density of 14/101 (13.8%) positive samples increased by > 50%, indicating that immune complexes likely form in many heartworm-infected dogs, but the degree to which the heartworm antigen is blocked varies [27]. A survey of dogs in animal shelters in Romania revealed that after heat pretreatment, 52/194 (26.8%) samples changed from negative to positive on antigen test [16]. Heat pretreatment also increases the number of antigen-positive cat samples. Evaluation of samples from shelter cats from the southern United States revealed 21/385 (5.5%) became positive after heat pretreatment; antibody to *D. immitis* was significantly more common in samples from cats that became antigen-positive after heat pretreatment than in samples that remained antigen-negative, supporting the interpretation that heat pretreatment was revealing true positives [32].

Reversal from antigen-negative to positive after pretreatment by heating also occurs in some pet dogs infected with heartworm. In one study, samples from 15 heartworm-infected pet dogs managed with either monthly ivermectin or monthly moxidectin preventive and an initial 30 day course of doxycycline, and that tested negative on an antigen test within 24 months of starting treatment, were re-evaluated to determine if this therapy had resulted in the development of false negative antigen tests. Indeed, “negative” samples from 8/15 (53.3%) dogs changed to positive after pretreatment [33]. In Brazil, samples from 22 pet dogs naturally infected with heartworms and managed with topical moxidectin/imidacloprid and doxycycline were evaluated. After 6 months of this therapy, samples from 6/14 (42.9%) dogs changed from negative to positive on the antigen test after heat pretreatment of the samples, and after 12 months, 1/21 (4.8%) changed to positive [34]. Interestingly, when the effect of moxidectin/imidacloprid treatment on adult *D. immitis* was evaluated in dogs experimentally infected *via* surgical transplantation of adult worms, pretreatment of serum with heating did not appear to provide additional sensitivity [35], a finding that may reflect the different host immune response invoked by transplantation of adult worms compared to natural or experimental infection by third-stage larvae. Heat pretreatment also proved helpful in confirming heartworm infection in a pet dog in Portugal co-infected with both *D. immitis* and *D. repens* [36] and was recently reported as helpful in resolving discordant results in a comparative evaluation of commercially available antigen tests [37].

Identified risk factors that appear to indicate samples from dogs in animal shelters are likely to change from negative to positive with heat pretreatment included concomitant presence of microfilariae and recent administration of a heartworm preventive [31]. In contrast, initial work shows that samples from pet dogs free of infection and maintained on heartworm preventives appear unlikely to become positive with heat pretreatment. Evaluation of samples from 201 pet dogs in Oklahoma (USA) for *D. immitis* antigen both before and after heat treatment revealed only one (0.5%) changed from negative to positive [38]. Although further research is needed, together these studies allow general recommendations to be made on which patients would benefit the most from heat pretreatment prior to antigen testing (Table 5).

**Table 5:** Patients^a^ most likely to benefit from heat pretreatment of samples prior to antigen testing for *Dirofilaria immitis*

Patient type	Reason
Dogs in endemic areas not on preventive, especially young dogs	Allows detection of infection earlier than testing non-pretreated samples.
Dogs with an inconsistent history of preventive use	Macrocyclic lactones given intermittently may begin to kill adult worms, leading to inflammation and immune complex formation. Microfilariae are also less likely to be detected in patients that have received preventives.
Heartworm-infected dogs recently treated with adulticide or managed only with preventive and doxycycline	Inflammation induced by dead and dying worms may lead to immune complex formation that masks antigen, preventing detection in unheated samples. Residual antigen may be detected both with and without heat pretreatment of samples in dogs recently treated for heartworm infection.
Dogs with microfilariae detected but that are antigen-negative	If microfilariae are *D. immitis*, then heat pretreatment is likely to change the result to true positive.
Cats in endemic areas not on preventive	Blocked antigen is very common in infected cats, particularly early in infection when immune response is most pronounced.

^a^Exercise caution in interpreting heartworm antigen test results, with or without heat pretreatment, on samples from dogs living in areas where infection with *Angiostrongylus vasorum* or *Spirocerca lupi* is common; both have been shown to cause false positive results on some antigen tests (see Table 8)

## Summary of data from diagnostic records

To explore this issue using clinical samples, we summarized the diagnostic records from 162 canine patients from the USA, including samples from Arkansas, California, Florida, Hawaii, Illinois, Oklahoma, Tennessee, Texas and Virginia. Each sample included in this review was tested for antigen before and after heat treatment and for microfilariae of *D. immitis* by modified Knott as previously described [26, 39]; additional samples tested from 12 other states but which did not have all three assays performed were excluded. All testing was performed at the Oklahoma Animal Disease Diagnostic Laboratory in 2017. For antigen testing, 1 ml of whole blood was centrifuged at 1500× *g* for 10 min, plasma removed and either tested directly (antigen test before heat) or heated to 104 °C for 10 min, the resultant coagulum centrifuged at 16,000× *g* for 10 min, and the supernatant tested (antigen test after heat) [26]. All antigen testing used a commercial assay according to manufacturer’s instructions (DiroCHEK®, Zoetis, Kalamazoo, Michigan, USA). For modified Knott test, 9 ml of 2% formalin was added to 1 ml of whole blood, the sample mixed by inversion, and then centrifuged at 1500× *g* for 10 min. The supernatant was decanted, the pellet stained with 2% methylene blue, and transferred to a glass microscope slide for examination [39]. Any microfilariae present were counted and the length and width of up to 10 individual microfilariae recorded.

Prior to heat treatment, 13.0% (21/162) of dogs were positive for *D. immitis*, including 14 by only antigen, six by only microfilaria detection, and one positive for both. After heat treatment, 24.7% (40/162) of dogs were positive for *D. immitis*, including 33 by only antigen. All six dogs that were microfilaria-positive for *D. immitis* but antigen-negative prior to heat treatment became antigen-positive following heat treatment (Table 6). All of these dogs were from Oklahoma and Texas, a finding that reflects the geographical distribution of most samples tested in this state diagnostic laboratory. Of the 17 dogs that changed from antigen-negative to antigen-positive after heat treatment but that did not have microfilariae detected, 16 were from Oklahoma and Texas and one was from Florida.

**Table 6:** Results of testing canine^a^ blood samples for antigen (Ag) of *Dirofilaria immitis* (*Di*) before and after heat pretreatment of samples and for microfilariae (MF) of *D. immitis* by modified Knott followed by morphological identification

Antigen test performed	Number (% positive)^b^	*Di* Ag + *Di* MF + (%)	*Di* Ag + *Di* MF - (%)	*Di* Ag - *Di* MF + (%)	*Di* Ag - *Di* MF - (%)
Before heat	21/162 (13.0)	1 (0.6)	14 (8.6)	6 (3.7)^c^	141 (87.0)^c^
After heat	40/162 (24.7)	7 (4.3)^c^	33 (20.4)^c^	0 (0)	122 (75.3)^c^

^a^Pet or shelter dogs, true heartworm status unknown

^b^Positive for *Dirofilaria immitis* by at least one laboratory method

^c^Three samples had microfilariae of *Acanthocheilonema reconditum*. The first contained both *D. immitis* and *A. reconditum* microfilariae and was negative before but positive after heat treatment. The second had only *A. reconditum* microfilariae and was negative before and negative after heat treatment. The third had only microfilariae of *A. reconditum* and was negative before and positive after heat treatment

This sample set does not represent a cross-section of all dogs tested for heartworm. Samples are more likely to be submitted to our diagnostic laboratory for testing when microfilariae are seen in a dog that tests antigen negative in clinic or when the veterinarian doubts the results of an antigen test based on history or physical examination. Nonetheless, the finding that the false negative antigen tests in microfilaremic dogs submitted to this laboratory reliably change to true positive with heat pretreatment is of interest. We do not know the true heartworm infection status of the 17 amicrofilaremic dogs whose samples changed to positive with heat pretreatment but suspect some of these may represent early, prepatent infections. Heat pretreatment can allow earlier detection of heartworm infection in both dogs and cats [28–30].

## Identification of microfilariae

Microfilariae of several species may be found in canine blood although the prevalence of each varies geographically. Commonly reported organisms include *Dirofilaria immitis*, *D. repens*, *A. reconditum* and *A. dracunculoides* [39–41]. All four organisms infect dogs in parts of Europe, Asia and Africa, but in the Americas, autochthonous canine infections of only *D. immitis* and *A. reconditum* have been reported [39, 41]. Diagnostic laboratories often identify microfilariae recovered on Knott test based on differences in morphology and size. For example, *D. immitis* is described as having a tapered head and straight body and tail, whereas *A. reconditum* bears a blunt head, curved body, and variably-shaped tail which may be either hooked or curved [39, 42]. Because multiple examples of ideal microfilaria are not present in every clinical sample, size measurements are also used as a primary criteria to identify the species present. Unfortunately, the length and width of each species varies widely among commonly used textbooks and other references (Table 7) and is also influenced by the fixation techniques used [43]. This variation can lead to misidentification of microfilariae, particularly when multiple *Dirofilaria* spp. or *Acanthocheilonema* spp. are present in a given area. In Romania, where co-infection with *D. immitis* and *D. repens* are common, identifying microfilariae by morphometric description alone can produce uncertain results [16, 43, 44].

**Table 7:** Reported length and width measurements of microfilaria of *Dirofilaria immitis, D. repens*^a^*, Acanthocheilonema reconditum,* and *A. dracunculoides*^a^ recovered by modified Knott (formalin fixed)

Measurement (μm)	*Dirofilaria immitis*	*Dirofilaria repens* ^a^	*Acanthocheilonema reconditum*	*Acanthocheilonema dracunculoides* ^a^	Reference
Length	295–325	268–360	250–288	189–230	[39]
	290–330	300–360	260–283	190–247	[42]
	295–308	359–380	259–270	253–266	[43]
	307–332	360	246–292	~ 300	[45]
	231–288^b^	302–344^b^	NR	NR	[46]
	≥ 315	NR	< 290	< 290	[68]
	280–320	NR	215–270	NR	[69]
Width	5.0–7.5	5.0–8.0	4.5–5.5	5.0–6.0	[39]
	5.0–7.0	6.0–8.0	4.0	4.0–6.5	[42]
	6.0–6.6	8.3–9.5	4.1–5.1	4.6–5.6	[43]
	6.8	12.0	4.7–5.8	NR	[45]
	6.1–7.2	NR	4.7–5.8	NR	[69]
	6.0–7.0	NR	< 5.6	NR	[70]

^a^Autochthonous infections not documented to occur in North America

^b^Microfilaria recovered by thick smear (methanol fixed)

Other approaches to microfilaria identification include histochemical stain using either acid phosphatase or Giemsa, and molecular approaches such as PCR with or without sequence confirmation. With acid phosphatase, microfilaria of *D. immitis* stains red at distinct focal points near the anal and excretory pore, *D. repens* stains only at the anal pore, *A. dracunculoides* stains at the anal pore, the excretory pore, and the internal body, while *A. reconditum* stains diffusely pink throughout [40, 45]. Using Giemsa stain, *D. immitis* is characterized by a longer cephalic space and the absence of distinct anterior nuclei when compared to *D. repens* [46]. Molecular identification has also commonly been used to confirm identity of microfilaria in blood with targets such as *cox*1, *12S* rDNA, and *16S* rDNA often employed [47, 48]. Unlike antigen testing, PCR can only detect patent infections when microfilariae are present in circulation; the main advantage of PCR over microscopic procedures such as the Knott test is that it allows confirmation of species by specific primers or sequencing.

## Additional explanations for false negative and false positive heartworm tests

False negative results, in which a dog or cat infected with heartworm does not have detectable antigen, can occur for a number of reasons other than antigen blocking. Until recently it was thought that antigen was not detectable until six months after infection, and that in dogs given heartworm preventives, antigen detection may be delayed as long as nine months after infection [1, 8]. More recent studies have documented that with heat pretreatment, antigen can be detected as early as 4.2 months in dogs or 5.6 months in cats [28–30]. In addition, recent work has shown that administration of preventives and doxycycline to an infected dog does interfere with antigen detection, but this false negative can be reversed with heat pretreatment of samples [33]. However, if dogs or cats harbor infections that have been established less than four or five months, respectively, immature *D. immitis* may be present and the antigen test negative regardless of how it is performed.

Testing platforms also differ in sensitivity. Infection with only one or two adult female heartworms may result in a negative patient-side assay but positive microtiter well result due to the greater sensitivity of the latter approach (Table 2, 3). Heat pretreatment has been shown to improve sensitivity of both patient-side and microtiter plate assays, and re-evaluation of test performance using pretreatment of samples was recently shown to resolve most discordant results [26, 37]. Finally, if only male worms are present, antigen is unlikely to be detected [4, 8]. The effect of heat pretreatment, if any, on detection of antigen in male-only infections has not been reported and such infections are considered uncommon and clinically unimportant.

The American Heartworm Society guidelines state that the current generation of heartworm antigen tests are “nearly 100% specific” and the label information on the assays and data from available comparative studies support this assertion (Table 2, 3) [8]. However, false positive results have been reported in samples from dogs and some wildlife species infected with nematodes other than *D. immitis* (Table 8). Importantly, false positives have been reported without any heat pretreatment of the samples [49–53]. Known causes of false positive heartworm antigen tests include infection with *Spirocerca lupi*, *Angiostrongylus vasorum* and *A. odendhali*, while related nematodes, such as other *Dirofilaria* spp., *Dracunculus insignis* and *Onchocerca* spp., are also thought to induce false positive results [49–53].

**Table 8:** Nematodes known or suspected to induce false positive results in canine serum, plasma, or whole blood samples tested on heartworm antigen tests without heat pretreatment of samples

Species of nematode	*Dirofilaria immitis* antigen detected (number positive/number tested)	Origin of samples tested	Reference
*Angiostrongylus vasorum*	0/16 to 8/16^a^	Domestic dogs experimentally infected with *An. vasorum*	[49]
*Spirocerca lupi*	2/19 to 14/48^a^	Domestic dogs^c^ with confirmed esophageal spirocercosis, resided in area without autochthonous *D. immitis*	[50]
	8/32	Domestic dogs^c^ with confirmed esophageal spirocercosis; PCR negative for *D. immitis* microfilaria	
*Acanthocheilonema odendhali*	15/15	California sea lions (*Zalophus californianus*) with microfilaria of *A. odendhali*	[51]
*Dirofilaria ursi*	6/10	North American brown bear (*Ursus arctos*) presumed infected based on geographic location (northern Canada)	[52]
*Angiostrongylus vasorum*	1/3 to 3/3^a^	Domestic dogs^c^ naturally infected with *An. vasorum* confirmed by serum antigen test for *An. vasorum* and fecal; history of consistent heartworm preventive use	[53]
*Dirofilaria repens*	0/4 to 2/4^a, b^	Domestic dogs^c^ naturally infected with *D. repens* as confirmed by microfilaria and subcutaneous nodules	[53]

^a^Results varied depending on heartworm antigen assay used

^b^With heat pretreatment of samples, increased to 4/4

^c^Pet dogs, true heartworm status unknown

In one recent publication, heat pretreatment was reported to reveal more false positives than was found using non-pretreated samples. Samples from three dogs with *An. vasorum* and four dogs with *D. repens* were tested with six different heartworm tests. Although positive antigen test results were evident prior to heat treatment, additional positives were detected after heat treatment [53]. Cross-reactions on *D. immitis* antigen tests have been previously documented in dogs infected with *An. vasorum* [49] but not *D. repens*. Co-infections with *D. immitis* and *D. repens* are commonly reported in areas where both occur [13, 16, 36, 54]; because all four dogs with *D. repens* in the cross-reaction paper were client-owned, necropsy results to support the absence of *D. immitis* were not available [53]. The effect of heat pretreatment on revealing additional false positives is not yet fully understood, but caution should be taken in interpreting results from samples pretreated prior to *D. immitis* antigen testing, particularly in populations likely to harbor infections with *An. vasorum* or *S. lupi*, nematodes known to cross-react on heartworm antigen tests.

## Conclusions

Heartworm antigen tests provide a convenient, sensitive, and specific means of identifying *D. immitis* infection in veterinary patients. However, recent research from multiple laboratories shows that false negative results may be present in many individual patients. For this reason, heat pretreatment of serum or plasma samples offers a valuable adjunct to traditional heartworm testing. As demonstrated in the present paper, this approach also allows resolution of *D. immitis* microfilaria-positive but antigen-negative results; in addition, heat pretreatment has been shown to resolve discordant results between different heartworm assays [37]. Although commonly described as virtually 100% specific, false positive results also have been reported and thus care should be taken in interpreting heartworm tests from dogs where autochthonous infections with *An. vasorum* or *S. lupi* occur regardless of whether heat pretreatment is performed.

## Abbreviations

*12S* rDNA: *12S* ribosomal RNA gene
*16S* rDNA: 16S ribosomal RNA gene
Ag: Antigen
*cox*1: Cytochrome *c* oxidase subunit 1
ELISA: Enzyme-linked immunosorbent assay
MF: Microfilaria
PCR: Polymerase chain reaction.
